# IntFOLD: an integrated server for modelling protein structures and functions from amino acid sequences

**DOI:** 10.1093/nar/gkv236

**Published:** 2015-03-27

**Authors:** Liam J. McGuffin, Jennifer D. Atkins, Bajuna R. Salehe, Ahmad N. Shuid, Daniel B. Roche

**Affiliations:** 1 School of Biological Sciences, University of Reading, Reading, RG6 6AS, UK; 2 Institut de Biologie Computationnelle, LIRMM, CNRS, Université de Montpellier, Montpellier 34095, France; 3 Centre de Recherches de Biochimie Macromoléculaire, CNRS- UMR 5237, Montpellier 34293, France

## Abstract

IntFOLD is an independent web server that integrates our leading methods for structure and function prediction. The server provides a simple unified interface that aims to make complex protein modelling data more accessible to life scientists. The server web interface is designed to be intuitive and integrates a complex set of quantitative data, so that 3D modelling results can be viewed on a single page and interpreted by non-expert modellers at a glance. The only required input to the server is an amino acid sequence for the target protein. Here we describe major performance and user interface updates to the server, which comprises an integrated pipeline of methods for: tertiary structure prediction, global and local 3D model quality assessment, disorder prediction, structural domain prediction, function prediction and modelling of protein-ligand interactions. The server has been independently validated during numerous CASP (Critical Assessment of Techniques for Protein Structure Prediction) experiments, as well as being continuously evaluated by the CAMEO (Continuous Automated Model Evaluation) project. The IntFOLD server is available at: http://www.reading.ac.uk/bioinf/IntFOLD/

## INTRODUCTION

The IntFOLD server is an integrated resource for modelling the structures and functions of proteins from sequences. Here we describe the significant major updates to the IntFOLD server, which was originally published in the NAR Web Server 2011 issue (1). The server has been operational since 2010, it has been used extensively by thousands of researchers around the world and processed tens of thousands of unique sequences. The server comprises an integrated suite of five novel methods: IntFOLD3-TS, for tertiary structure prediction; ModFOLD5, for model quality assessment; DISOclust3, for disorder prediction; DomFOLD3, for domain prediction and FunFOLD3, for function and ligand binding site prediction.

The component methods within the IntFOLD server form a single sequence-structure-function annotation pipeline, with the data output from one algorithm forming the input data for another. Such integration increases the efficiency of computation and server management, and reduces the time researchers will have to spend submitting predictions and collating and interpreting their results. Alternative servers are available that generate results using related individual methods, however, the IntFOLD server is perhaps unique in providing an integrated pipeline of multiple methods with class leading model quality assessment algorithms built-in. In addition, the server provides a single results page with unified graphical output and a single point of entry for job submission, allowing ease of access to non-experts. Furthermore, the server provides links to all of the raw machine readable output data in the standard CASP formats, for use by expert developers and independent assessors.

The individual algorithms for the IntFOLD server components have been previously described and benchmarked (2–5). In this paper we focus on the major modifications to each of these component algorithms and their integration into the IntFOLD server, which have led to successive performance gains since the original publication describing the server. Furthermore, we report on the provision of new structural and functional data outputs as well as several user interface improvements.

## MATERIALS AND METHODS

### Tertiary structure prediction using IntFOLD3-TS

The original IntFOLD-TS method used a single-template local consensus fold recognition approach to predict protein tertiary structure from sequence (6). The method has since been updated to use a novel multiple-template modelling approach that is guided by global and local quality estimates (3). The latest version further improves model accuracy through inclusion of additional sequence-structure alignment methods (7,8). Clear progress in modelling accuracy can be seen over the successive versions of IntFOLD according to independent benchmarks (9–11) (Supplementary Table S1).

### Model quality assessment using ModFOLD5

Model quality assessment scores help us to distinguish, with confidence, those specific regions of a 3D protein model that might be untrustworthy from those that are close to reality. Importantly, accurate quality checking scores are built directly into the 3D models using our latest version of ModFOLD. ModFOLD5 adopts a quasi-single model approach for quality estimation, which has been described previously (5), however with our latest approach, a greater number and variety of reference models are generated using the IntFOLD3-TS pipeline described above. The ModFOLD components of the IntFOLD server have been independently benchmarked, performing favourably in comparison with other servers (9,12–13) (Supplementary Table S2)

### Domain prediction using DomFOLD3

The DomFOLD3 method utilizes the Protein Domain Parser (PDP) method (14) in order to identify the independent folding units (domains) in the top model obtained from the IntFOLD3-TS method. The output from PDP is then parsed to produce per-residue domain assignments, provided both in CASP format and utilizing the B-factor column in the model file. This approach has been benchmarked in previous CASP experiments (15,16); however the category of domain prediction has since been removed by the CASP organizers.

### Prediction of intrinsically disordered regions using DISOclust3

Many proteins have regions of structural instability or intrinsic disorder. It is useful to identify these regions for further laboratory studies as they are difficult to resolve experimentally. Furthermore, disordered regions are often functionally relevant. The DISOclust3 method analyses the ModFOLDclust2 (17) per-residue scores in order to identify the regions of high variability occurring in the multiple alternative 3D models that are generated by the IntFOLD3-TS method. The mean per-residue scores are then combined with those from DISOPRED3 (18) to form a final prediction. The method has performed well in previous CASP experiments (19–21), but the category was cancelled in CASP11 due to lack of suitable targets.

### Prediction of protein–ligand interactions using FunFOLD3

Structure predictions can be used to provide crucial information about a protein's function. The FunFOLD algorithm works by carrying out model-to-template superpositions, of the top ranked IntFOLD3-TS 3D models and related templates with bound ligands, in order to identify the 3D locations of binding site residues and putative interacting ligands (22). An agglomerative hierarchical clustering algorithm is used for identifying putative ligands and a voting system is used for residue selection. The FunFOLD approach has performed well in recent CASP experiments (23,24). The method has since been updated to include a range of new binding site quality estimates (25) as well as per-atom *P*-values for ligand contacts in CAMEO-LB format (4). The latest version of the method also makes use of BioLip (26) data to determine if ligands are biologically significant at multiple sites as well as providing functional annotations as EC numbers and GO terms.

## RESULTS

### Server inputs and outputs

#### Inputs

The only required input to the server is an amino acid sequence for the target protein. However, users may optionally provide a name for their protein sequence, their email address and alternative 3D models of their protein, for direct comparison and quality assessment.

#### Graphical outputs

**Figure 1.**
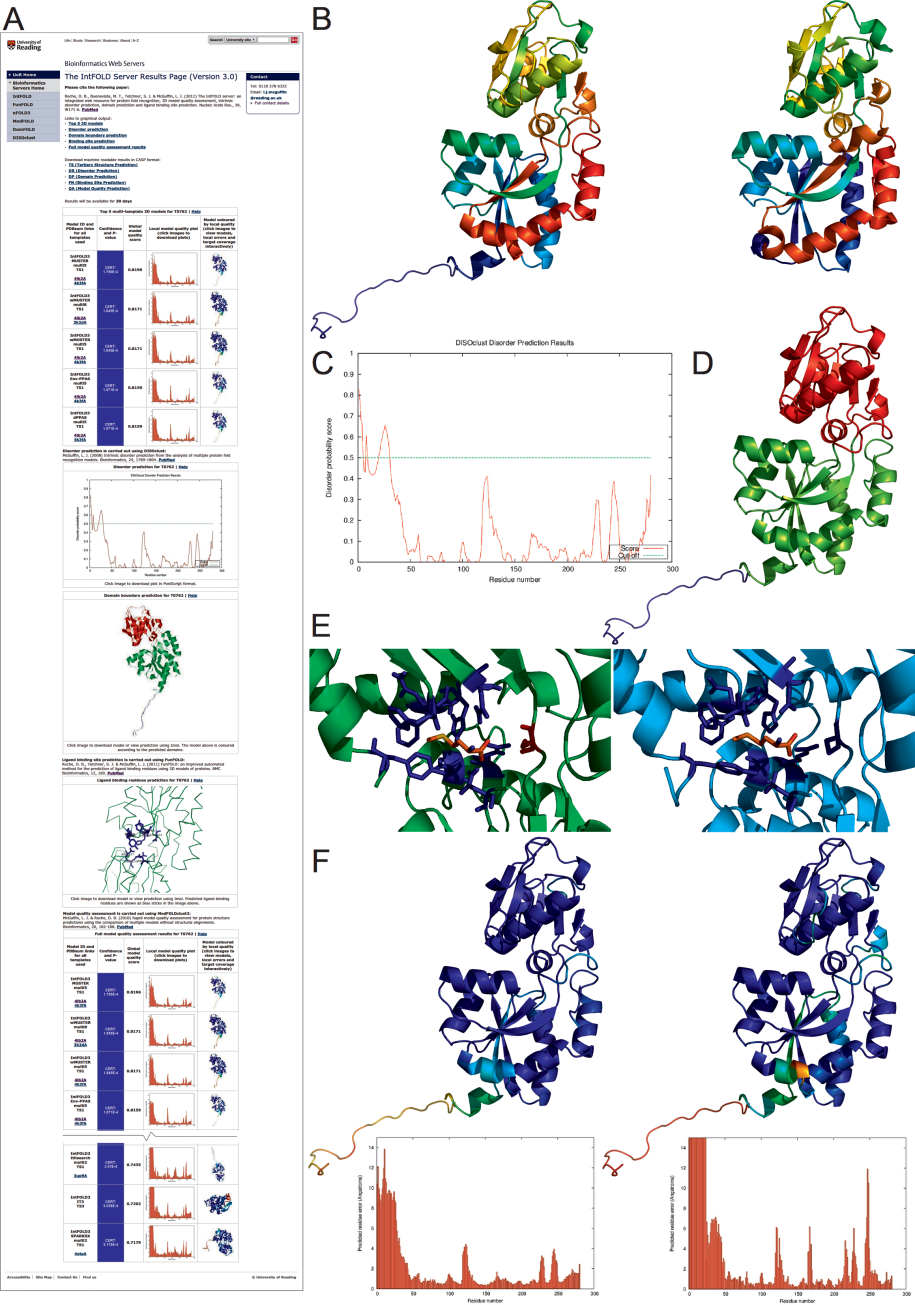
IntFOLD server results for an example protein target from CASP11 (T0762; PDB ID: 4q5t) (**A**) An example of the graphical output from the server showing the main results page with a summary of the results from each method. The page starts with the top five 3D models followed by the disorder prediction, domain prediction, ligand binding site prediction and the full model quality results (truncated here to fit page). Clicking on the model images leads to interactive views of models, which can be manipulated in 3D using the JSmol/HTML5 framework and/or downloaded for local viewing. (**B**) Predicted 3D model (left) and observed (right) tertiary structures are compared using the spectrum colouring scheme (TM-score(27) = 0.92). (**C**) The disorder prediction plot with residue number on the x-axis and disorder probability on the y-axis. (**D**) The structural domain prediction is mapped onto the top 3D model—blue: domain1 (disordered), green: domain2, red: domain 3. (**E**) The top predicted 3D model (left, green) and observed structure (right, cyan) with binding site residues and ligands. The correctly predicted binding site residues [69, 86, 87, 88, 91, 147, 150, 206, 235] are shown as blue sticks and the predicted ligand (MET) is coloured by element. The only under-predicted binding residue [112-SER] is coloured red. The binding site prediction has an MCC score of 0.9468 and a BDT score (29) of 0.9000. (**F**) Model quality assessment results for the top 3D model. Predicted model quality (left) is compared with observed model quality (right). In the left image the blues and greens represent residues predicted to be closer to the native structure, while oranges and reds represent those that deviate from (or are missing in) the native structure. The right image shows the actual results for the model when compared with the native structure using the same colouring scheme. Below each image are the predicted (left) and observed (right) per-residue error plots with the residue number on the x-axis and the predicted residue error (distance of the Cα atom from the native structure in Ångstroms) on the y-axis. Correlation analysis of the targets suggest that there is a strong positive correlation between the observed and predicted residue scores (Pearson's *R* = 0.917, Spearman's rho = 0.772, Kendall's tau *B* = 0.588). The images in B, D, E and F were rendered using PyMOL (http://www.pymol.org/).

The output is presented as a simple table that summarizes all prediction data graphically through thumbnail images of plots and annotated 3D models (Figure 1A). A detailed help page is provided, which serves as a guide for interpreting results with links to several example results pages. The help page also includes information on the input data formatting and detailed descriptions of the output data from the server. Users can click through the images in the table in order to drill down into individual results, download the 3D coordinates of models in PDB format and/or directly view them interactively in 3D using the new JSmol/HTML5 framework (http://www.jmol.org/). In previous versions of the IntFOLD server, the Java plugin was used, however with the new HTML5 version, interactive results are available to view using any modern browser, including on tablets and mobiles, without the need for plugin installation. Numerous useful improvements to the results pages have been provided since the original publication, such as: *P*-values for global 3D model quality scores; interactive displays showing the structural superpositions of templates and models; new scores and *P*-values for estimating the quality of ligand binding; EC numbers and GO terms for functional annotation; and better error handling and job status reporting is now provided to the user.

The images in Figure 1B–F serve to demonstrate results obtainable from each of the integrated methods. In Figure 1B, the predicted IntFOLD3-TS model for CASP11 target T0762 is shown to be significantly similar to the observed structure, with the TM-score of 0.92 (27). The IntFOLD3-TS model provides coordinates for the full length sequence including the short N-terminal disordered region, which is not visible in the crystal structure. In Figure 1C, the disorder prediction plot from DISOclust3 also indicates that the first few residues of the protein are unstructured. The discontinuous structural domains assigned by DomFOLD3 are highlighted in Figure 1D. For CASP11, target T0762 was assessed as a single domain, however related templates in CATH (28) are shown to have the same discontinuous domain organization as was predicted. In Figure 1E, the FunFOLD3 predicted ligand binding site shows a close match compared with the observed protein–ligand interaction, where the MCC (Matthews Correlation Coefficient) and BDT (Binding site Distance Test) scores (29) are ≥ 0.9. Finally, in Figure 1F the predicted and observed local quality scores of the top predicted 3D model are also shown to correlate significantly (see also Supplementary Figures S1 and S2).

#### Machine readable outputs

The raw machine readable data files for each set of predictions are also provided for developers, which comply with the CASP data standards and the new formats required by the CAMEO project.

### Independent benchmarking

Each major version of the IntFOLD server has been independently tested in the CASP9-CASP11 experiments and its component algorithms have often performed well, ranking among the top independent servers in the tertiary structure, quality assessment, disorder and function prediction categories. The IntFOLD server has performed exceptionally well in the quality assessment category, outperforming most other servers in the QA3 self-assessment or ‘B-factor’ predictions (10,12) (http://www.predictioncenter.org/casp11/doc/presentations/CASP11_QA_AK.pdf). The server recently become a partner site of the protein model portal (30) for interactive 3D modelling and is continuously benchmarked by CAMEO (http://www.cameo3d.org). Successive improvements have been observed in the subsequent versions, since the original version published in 2011. A summary of CAMEO results from the last 6 months for the latest and previous IntFOLD server methods are shown in Supplementary Tables S1 and S2.

## DISCUSSION

The IntFOLD server provides an accessible and unified interface to our leading methods for the prediction of protein structures and functions from amino acid sequences. The server provides a clean web interface that integrates a complex set of quantitative prediction data, producing a graphical summary of results that may be easily interpreted by non-expert predictors. The algorithms underlying the IntFOLD server have been extensively independently tested and found to be competitive in several prediction categories by the CASP assessors and by the CAMEO project. One major distinguishing feature of the IntFOLD server 3D models is that they include high accuracy per-residue quality estimations built-in to the B-factor column of the coordinate files. The IntFOLD server has been pioneering and is a class leader in this respect, which has helped to influence and encourage competing developers to follow suit. However, the IntFOLD server still outperforms many other servers in terms of the accuracy of its self-assessment. Finally, in addition to continual improvements to accuracy of the IntFOLD sequence-structure-function prediction pipeline, we have also made improvements to the server stability, the job status reporting and the user interface since the original server.

## SUPPLEMENTARY DATA

Supplementary Data are available at NAR Online.

SUPPLEMENTARY DATA
