# Supplementary material for: IntFOLD: an integrated server for modelling protein structures and functions from amino acid sequences
Source: Nucleic Acids Res. 2015 Mar 27;43(Web Server issue):W169–73. doi: 10.1093/nar/gkv236 (PMC4489238; doi:10.1093/nar/gkv236)
Supplement: SUPPLEMENTARY DATA [file supp_43_W1_W169__index.html]

IntFOLD: an integrated server for modelling protein structures and functions from amino acid sequences — SUPPLEMENTARY DATA 

# IntFOLD: an integrated server for modelling protein structures and functions from amino acid sequences

## SUPPLEMENTARY DATA

**Files in this Data Supplement:**

- SUPPLEMENTARY DATA
