# Supplementary material for: IntFOLD: an integrated server for modelling protein structures and functions from amino acid sequences
Source: Nucleic Acids Res. 2015 Mar 27;43(Web Server issue):W169–73. doi: 10.1093/nar/gkv236 (PMC4489238; doi:10.1093/nar/gkv236)

Supplementary Table 1. CAMEO (9) pairwise comparison of 3D modelling servers. Dataset from last 6-months (2014-07-18 to 2015-01-09). IntFOLD3-TS (server33) is the reference server compared against older IntFOLD versions and other public servers on common targets.

Server Name	Number of models in common subset	Avg. lDDT difference*	Avg. lDDT-Cα difference**	Avg. lDDT binding sites difference***	Avg. GDT_HA difference*
Robetta	220	-4.4	-3.88	-0.02	-3.43
RaptorX	216	-1.14	-1.12	**0.01**	-0.67
IntFOLD2-TS	214	**0.17**	**0.07**	0	-0.05
RBO Aleph	186	**1.19**	**1.68**	**0.02**	**2.94**
M4T	120	**1.74**	**1.97**	**0.03**	**1.66**
IntFOLD-TS	208	**3.56**	**3.75**	**0.02**	**3.7**
SWISS-MODEL	229	**4.32**	**4.8**	-0.01	**1.53**
Princeton_TEMPLATE	215	**4.71**	**1.88**	**0.11**	**2.27**
HHpredB	206	**6.85**	-1.01	-0.05	-0.56
NaiveBlits	119	**7.87**	**9.08**	**0.01**	**3.99**
NaiveBLAST	203	**10.67**	**12.43**	**0.06**	**6.51**
Phyre2	172	**15.16**	**11.75**	**0.07**	**6.41**

The lDDT score is the local Distance Difference Test on all atoms that assess the quality of the local atomic environment of a model. The lDDT acts as an incentive scheme for correctly predicted interatomic distances in a model at different threshold levels. If the lDDT score difference* is below the given threshold level, the interaction between interatomic distances is considered to be preserved in the prediction. The final lDDT-all score is computed by averaging the fraction of correctly modelled interactions for the following four distance difference thresholds: 0.5, 1, 2, and 4 Å. This is the same as GDT_HA which identifies collection of residues that are away from the target but not more than stated Cα distance thresholds** for varying superposition. The Avg. lDDT score*** is limited to those residues which form the binding site(s) on the respective target. Source of data and information on scoring methods: http://www.cameo3d.org/.

Supplementary Table 2. CAMEO (9) model quality estimation performance. Data from last 6-months (2014-07-18 to 2015-01-09). ModFOLD5 (server 9) and ModFOLD4 comparison with other methods.

Predictor Name	Number of models processed (out of 1935 submitted)	**ROC**		**ROC normalised**		**PR**		**PR normalised**	
		$AUC_{0,1}$	$AUC_{0,0.2}$	$AUC_{0,1}$	$AUC_{0,0.2}$	$AUC_{0,1}$	$AUC_{0.8,1}$	$AUC_{0,1}$	$AUC_{0.8,1}$
ModFOLD5 (Server 9)	1837	0.86	**0.59**	**0.82**	**0.56**	0.79	0.54	**0.75**	**0.51**
ModFOLD4	1745	**0.87**	**0.59**	0.78	0.53	**0.8**	**0.55**	0.72	0.49
Qmean 7.11	1927	0.82	0.5	0.81	0.5	0.74	0.47	0.74	0.47
ProQ2	1659	0.84	0.57	0.72	0.49	0.79	0.49	0.68	0.42
Verify3d smoothed	1935	0.71	0.34	0.71	0.34	0.62	0.4	0.62	0.4
Dfire v1.1	1935	0.66	0.25	0.66	0.25	0.53	0.39	0.53	0.39
Naive PSIBlast	1928	0.66	0.25	0.65	0.25	0.53	0.39	0.53	0.39

The ROC (Receiver Operating Characteristics) and PR (Precision and Recall) scores in bold indicate the highest prediction score of the ModFOLD5/4 comparing to other predictors. The $AUC_{0,1}$ (area under the curve) is the range from 0 to 1 (0 – perfect predictions and 0.5 – random prediction) that describes True Positive Rate (TPR) and False Positive Rate (FPR) computed for all positives thresholds. $AUC_{0,0.2}$ is the partial AUC of the ROC 'trimmed' threshold of 0.2 and scaled between 0 and 1.$ROC_{normalised}$ is the same scale as ROC but has been normalised. PR curve analysis is an alternative to ROC for task with large skew in the class distribution and it helps highlighting differences in the predictor performances that are not clear in the ROC. $PR_{normalised}$ is the same metric of the column PR with the values normalised by the submitted/received target ratio. Source of data and information on scoring methods: http://www.cameo3d.org.

Supplementary Figure 1. Line plot showing the overlay of the predicted and observed per-residue errors in the top IntFOLD3-TS model for CASP target T0762.

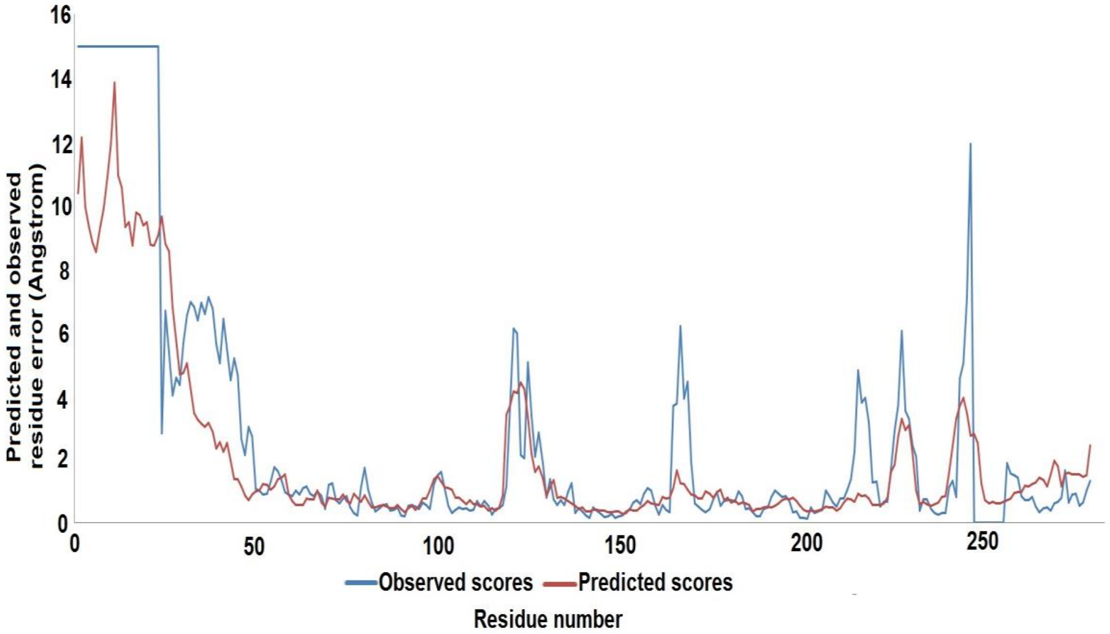

Supplementary Figure 2. Scatter plot and correlation analysis of the predicted and observed per-residue errors in the top IntFOLD3-TS model for CASP target T0762. The plot shows a strong positive correlation between the observed and predicted residue scores: Spearman's rho (0.772), Kendall's tau B (0.588), and Pearson's R (0.917). The observed versus predicted correlation test indicated that there is a significant (P<0.001) positive correlation between the observed and predicted scores.

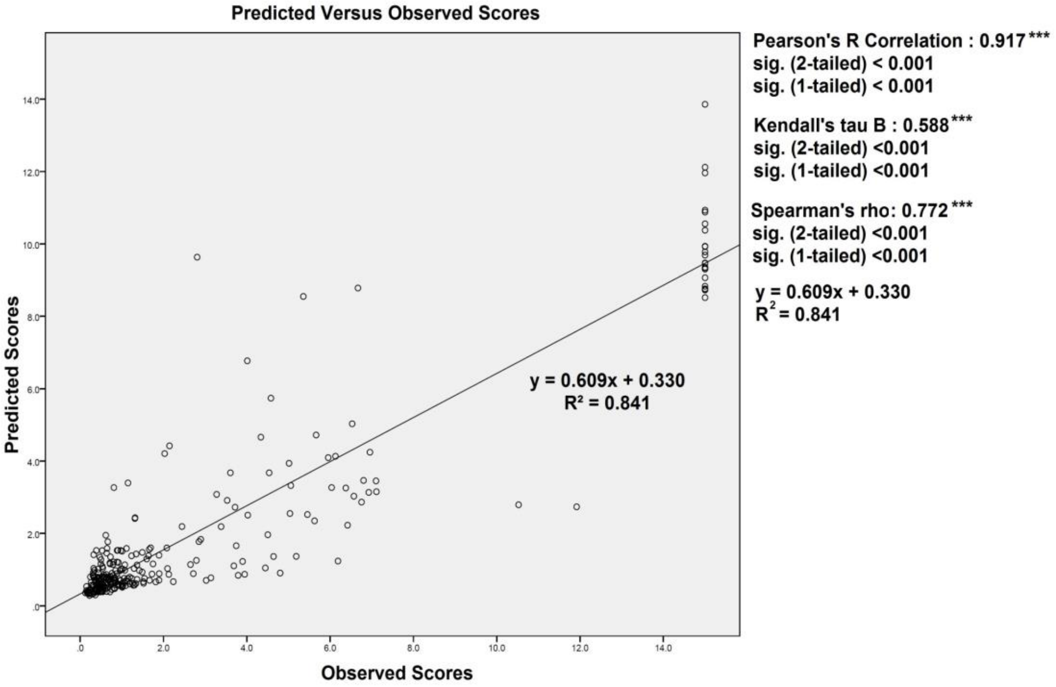

Supplement: SUPPLEMENTARY DATA [file supp_gkv236_nar-00305-web-b-2015-File003.pdf]
